# Recombinant protein production in *Pseudoalteromonas haloplanktis* TAC125 biofilm

**DOI:** 10.1016/j.bioflm.2024.100179

**Published:** 2024-01-24

**Authors:** Marzia Calvanese, Caterina D'Angelo, Concetta Lauro, Maria Luisa Tutino, Ermenegilda Parrilli

**Affiliations:** a Department of Chemical Sciences, University of Naples “Federico II”, Complesso Universitario Monte S. Angelo, Via Cintia 4, 80126, Naples, Italy; b Istituto Nazionale Biostrutture e Biosistemi I.N.B.B, Viale Medaglie D’Oro, 305-00136, Roma, Italy

**Keywords:** Biofilm, *Pseudoalteromonas haloplanktis* TAC125, Recombinant protein production, Cold-adapted bacteria, m-Scarlet, GFP

## Abstract

Biofilms have great potential for producing valuable products, and recent research has been performed on biofilms for the production of compounds with biotechnological and industrial relevance. However, the production of recombinant proteins using this system is still limited. The recombinant protein production in microbial hosts is a well-established technology and a variety of expression systems are available. Nevertheless, the production of some recombinant proteins can result in proteolyzed, insoluble, and non-functional forms, therefore it is necessary to start the exploration of non-conventional production systems that, in the future, could be helpful to produce some “difficult” proteins. Non-conventional production systems can be based on the use of alternative hosts and/or on non-conventional ways to grow recombinant cells. In this paper, the use of the Antarctic marine bacterium *Pseudoalteromonas haloplanktis* TAC125 grown in biofilm conditions was explored to produce two fluorescent proteins, GFP and mScarlet. The best conditions for the production were identified by working on media composition, and induction conditions, and by building a new expression vector suitable for the biofilm conditions. Results reported demonstrated that the optimized system for the recombinant protein production in biofilm, although it takes longer than planktonic production, has the same potentiality as the classical planktonic approach with additional advantages since it needs a lower concentration of the carbon sources and doesn't require antibiotic addition. Moreover, in the case of mScarlet, the production in biofilm outperforms the planktonic system in terms of a better quality of the recombinant product.

## 1 Introduction

During the past few decades, considerable progress has been made in microbial platform engineering to improve the productivity and yields of recombinant proteins [[1], [2], [3]]. All advancements in this area have increased and facilitated the recombinant protein production in several organisms [4,5]. Although a variety of expression systems, vectors, and host strains are available (*E.coli, Bacillus subtilis, Saccharomyces cereviae, Pichia pastoris* etc …), the production of some recombinant proteins can still result in heavily proteolyzed, insoluble, and non-functional forms [1]. These phenomena can be attributed to several factors and although various strategies have been applied to overcome these limitations, there is no guarantee that every type of recombinant protein will have a high production yield or catalytic/functional activity. Therefore, it is necessary to explore and consolidate the use of non-conventional production systems that could be useful in producing some “difficult” proteins. Non-conventional production systems can be based on the use of alternative hosts and/or on non-conventional ways to grow the cells for recombinant protein production. In this paper, we explored the use of a non-conventional host, the Antarctic marine bacterium *Pseudoalteromonas haloplanktis* TAC125 (*Ph*TAC125) [6], grown in biofilm conditions to produce recombinant proteins.

*Ph*TAC125 genome was fully sequenced and annotated [7] and it is currently exploited as a new alternative expression host, its physiological features including fast growth in a wide range of temperatures and efficient protein synthesis make *Ph*TAC125 an attractive and versatile host to produce recombinant proteins [8,9]. Several expression vectors have been developed to allow the production of several recombinant proteins either by constitutive or inducible promoters [[9], [10], [11], [12]]. The advantages in using these cold-adaptive systems in alternative to the conventional hosts were proven in the production of some “difficult proteins” [[13], [14], [15], [16]].

Most of the research on the production of recombinant proteins has been performed on cells grown in planktonic cultures. However, biofilm is the most successful and widely distributed form of life on earth [17]. It is not simply structured assemblages of cells but a dynamic complex system that evolves through a tightly controlled multistep process [18]. The first step is characterized by a loose/transient association [19], followed by robust adhesion [20] and colonization, where microorganisms are attached to the surface or interface via stronger interactions, and maturation [21]. The last stage is characterized by a return to transient motility where biofilm cells are detached, due to either intrinsic or extrinsic factors, and disseminated cells colonize other sites [22]. Biofilm provides bacteria characteristics deeply different from their planktonic counterparts [[23], [24], [25]], mainly derived by the genetic and metabolic rewiring of the biofilm-dwelling bacteria [26]. The local environmental conditions arising within the biofilm matrix, the intercellular signaling, and other phenomena, may induce cells in biofilm to modulate the expression of genes differently than in planktonic populations [25]. Moreover, biofilms are known to provide a fitness advantage under stress and harsh conditions [27], and this property could be useful to alleviate the host metabolic burden associated with heterologous protein overproduction. Furthermore, the sessile growth may allow for reaching high biomass concentration, indeed, it has been reported that biofilm reactors can retain 5-10 times more biomass *per* unit volume with respect to canonical one [28], and the high plasmid maintenance in biofilm [29,30] could guarantee good operation stability. All these properties could be very useful in industrial processes aimed at recombinant protein production. Although several potential drawbacks exist regarding the use of biofilm in recombinant protein production, mainly related to the control of biofilm development, this non-conventional process has several interesting potentials that justify additional studies, so much so that different studies explored this production approach in several microorganisms [31,32]. The first system for high level heterologous protein production biofilm cells was aimed at the production of eGFP in *E. coli* [33]. These authors demonstrated that the biofilm environment enhanced plasmid maintenance and the studies of Mergulhao and coworkers [28,32] showed that the GFP production levels were higher levels than their planktonic counterparts. Moreover, recently *E. coli* biofilm was successfully used to produce the human epidermal growth factor (hEGF) in a continuous process [31].

In this paper, we explored the feasibility of this alternative methodology in *Pseudoalteromonas haloplanktis* TAC125 (*Ph*TAC125). This Antarctic marine bacterium is a promising unconventional host for the production of high-value proteins, thanks to its physiological features: fast growth in a wide range of temperatures and efficient protein synthesis [6,34]. Furthermore, for this bacterium an efficient gene expression technology was established [9,[35], [36], [37]] and either constitutive or inducible systems [11,38,39] were developed. The implementation of this psychrophilic system [40], the availability of engineered strains [11,41] and the formulation of suitable synthetic media [38] allowed the production of difficult-to-express proteins in an active and soluble form [42,43]. Additionally, *Ph*TAC125 forms biofilm either when grown in rich or synthetic medium, and the quantity of biofilm produced in the synthetic medium GG was higher [44]. The analysis of *Ph*TAC125 biofilm structure revealed the presence of different sugars which are characteristic of LPS but also, *N*- Acetyl-muramic acid (MurNAc, NAM), ribose, glucose, and mainly cellulose [45], this polymer is a common component of biofilms and mediates cell-cell interactions and cell adherence on surfaces [46]. Starting from the knowledge of *Ph*TAC125 biofilm features and from the availability of different tools for recombinant protein production in this alternative host, we explored the production of recombinant proteins in biofilm. After the assessment of this option by the production of two different fluorescent proteins (GFP and mScarlet), the optimization of the production process was faced by working on media composition and by building a new expression vector suitable for the biofilm conditions.

## 2 Materials and methods

### 2.1 Bacterial strains, media, and plasmids

The strains, plasmids, and oligonucleotides used in this study are listed in Table S1. *E. coli* TOP10 was used for cloning purposes, while *E. coli* S17-1 (*λpir*) was employed in intergeneric conjugations as a donor strain for *Ph*TAC125 transformations. *E. coli* strains were grown in Lysogen broth (LB, 10 g/L bacto-tryptone, 5 g/L yeast extract, 10 g/L NaCl) at 37 °C with 220 rpm. When required, 34 μg/mL chloramphenicol (Merck, Darmstadt, Germany) was supplemented to the medium. *Ph*TAC125, KrPL, and KrPL *LacY* + strains were tested to assess the kinetic of biofilm formation. The KrPL *LacY* + strain was used for the expression of the GFP and mScarlet proteins. On the other hand, the resulting constitutive expression vectors, pAT-2620-*mScarlet*, pAT-2621-*mScarlet*, and pAT-2690-*mScarlet*, were conjugated in KrPL. The psychrophilic strains were grown in the TYP medium (16 g/L bacto-tryptone, 16 g/L yeast extract, 10 g/L NaCl) during interspecific conjugations and precultures development. The first trials of recombinant protein production in biofilm and planktonic conditions were carried out at 15 °C in the synthetic medium GG (10 g/L l-glutamic acid monosodium salt monohydrate, 10 g/L d-gluconic acid sodium salt, 10 g/L NaCl, 1 g/L NH_4_NO_3_, 1 g/L K_2_HPO_4_, 200 mg/L MgSO_4_·7H_2_O, 5 mg/L FeSO_4_·7H_2_O, 5 mg/L CaCl_2_) [47], and 25 μg/mL chloramphenicol. Afterwards, the recombinant production was enhanced by growing the psychrophilic strains in the GG medium with half the carbon sources (named 5/5 GG) and supplemented with 70 mg/L FeSO_4_.

### 2.2 Construction of the expression plasmids

**Fig. S1:**
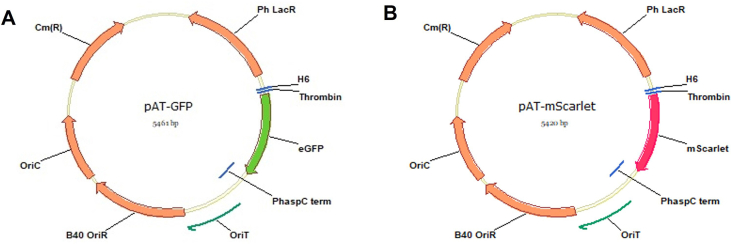
Maps of pAT-*gfp* and pAT-*mScarlet* vectors.

The DNA fragment containing the Shine-Dalgarno and spacing sequences of the *Ph*TAC125 *trpA* gene was synthesized by Thermo Fisher Scientific and cloned into pB40-79BsC [41], a high-copy number derivative of p79C [11], using *Bsa*I and *Kpn*I restriction sites. The resulting vector was named pAT. The genes encoding the fluorescent proteins, GFP and mScarlet, were introduced in pAT using *Nde*I and *Sac*I restriction enzymes, obtaining pAT-*gfp* and pAT-*mScarlet* (Fig. S1). Such vectors were mobilized in KrPL *LacY* ^*+*^ by conjugation [48].

The constitutive plasmids pAT_2620-*mScarlet*, pAT_2621-*mScarlet*, and pAT_2690-*mScarlet* were constructed by replacing the regulatory elements *Ph*TAE79 *lacR-lacZ* of pAT-*mScarlet* with the putative promoter sequences of three genes in *Ph*TAC125 genome (*PSHAa2620*, PSHAa2621, and PSHAa2690). The primers (PSHAa262*0_SphI* Fw, *PSHAa2620_NcoI* Rv, *PSHAa2621_SphI* Fw, *PSHAa2621_NcoI* Rv, *PSHAa2690_SphI* Fw, and *PSHAa2690_NcoI* Rv) listed in Table S1 were used to amplify a specific genomic sequence upstream of the selected genes (682 bp for PSHAa2620 and 410 bp for both PSHAa2621 and PSHAa2690). The PCR reaction was performed in a volume of 50 μL containing 4 ng of *Ph*TAC125 genomic DNA as template, 0.5 μM primers, 1 × HF Phusion buffer (New England Biolabs, Hitchin, UK), 200 μM of each dNTP, and 0.02 U/μL Phusion DNA Polymerase (New England Biolabs, Hitchin, UK). The PCR amplified fragments were double digested with *Sph*I/*Nco*I restriction enzymes and cloned into pAT-*mScarlet* previously digested with the same restriction sites. The new vectors (pAT_2620-*mScarlet*, pAT_2621-*mScarlet*, and pAT_2690-*mScarlet*) were mobilized into KrPL by conjugation.

### 2.3 Growth of planktonic and biofilm cultures

To evaluate the biofilm formation, the wild-type (*Ph*TAC125, KrPL, KrPL *LacY*^*+*^) and the recombinant strains (KrPL *LacY* + pAT-*gfp*, KrPL *LacY* + pAT-*mScarlet*) were grown in the GG or 5/5 GG media in static conditions. The chloramphenicol was used as a selective agent at a final concentration of 25 μg/mL when specified.

The KrPL pAT_2620-*mScarlet,* KrPL pAT_2621-*mScarlet*, and KrPL pAT_2690-*mScarlet* strains were grown in 5/5 GG without antibiotics and in presence of different iron sulfate concentrations (0.5 mg/L FeSO_4_, 5 mg/L FeSO_4_ the concentration used in the GG medium, 70 mg/L FeSO_4_) in static conditions for different times (24 h, 48 h, 72 h, 96 h).

For recombinant production in biofilm, the recombinant strains were grown by a fluidized-bed reactor using floating polystyrene supports with a total surface area of 12 cm^2^ [49] in the GG medium supplemented with chloramphenicol (25 μg/mL) starting from 0.2 OD_600nm_. The recombinant expression was induced at the beginning of growth (0.2 OD_600nm_) with 5 mM IPTG and incubated in a static condition at 15 °C. Afterwards, the growth conditions in the biofilm were optimized, reducing the carbon sources (5/5 GG), increasing the iron sulfate concentrations (70 mg/L FeSO_4_), and avoiding the use of chloramphenicol. The cells were harvested after 96 h and sonicated twice at a constant ultrasound frequency of 37 kHz for 15 min to allow biofilm detaching and biomass recovery from the polystyrene supports. After sonication, the bacterial cultures were centrifuged at 6′000 rpm for 30 min at 4 °C. Then the supernatant was discarded, and cell pellets were stored at −20 °C for the following analysis.

For planktonic cultures, the recombinant strain KrPL pAT_2620-*mScarlet* was grown in the 5/5 GG medium shaking at 15 °C and the culture was harvested after 72 h. The recombinant strain KrPL *LacY* + pAT-mScarlet was grown in the GG medium supplemented with 70 mg/L FeSO_4_ and chloramphenicol (25 μg/mL) and was induced during the exponential growth phase (1.0–1.5 OD_600nm_) with 5 mM IPTG, the culture was harvested after 72 h from the induction.

### 2.4 Cell lysis

To evaluate the mScarlet production in KrPL pAT_2620-*mScarlet*, the cell pellets (about 220 mg) were recovered from planktonic and biofilm growths after 72 h and 96 h, respectively, and resuspended in 30 mL lysis buffer (50 mM sodium phosphate pH 7.75, 500 mM NaCl, one tablet of EDTA-free Complete Ultra protease inhibitor (Roche, Mannheim, Germany). The planktonic cells were mechanically lysed by a French Press at 2 Kbar for two consecutive cycles; instead, five steps at 2.5 Kbar were used to disrupt the biofilm cells. The obtained lysates were centrifuged (6′500 rpm (4732 *g*) for 1 h at 4 °C) to separate the soluble and insoluble protein fractions, and then the insoluble fraction was resuspended in 30 mL PBS.

### 2.5 Fluorescence assay

The fluorescence intensity of GFP or mScarlet was measured using a JASCO FP-750 spectrofluorometer (Jasco Corp., Japan) at 25 °C with a 1 cm path length. Cell pellets (about 1,5 mg) were recovered at the end of the growths in planktonic or static growth conditions by centrifugation (13′000 rpm for 10 min at 4 °C), resuspended in 0.5 mL PBS, and serially diluted to achieve the best signal-to-noise ratio in fluorescence measurements. The excitation wavelength was set at 488 nm, and the intensity of emitted fluorescence of GFP at 507 nm was recorded [50]. Regarding mScarlet, the excitation and emission wavelengths were set at 569 and 690 nm, respectively [51]. Fluorescence intensities are reported in arbitrary units (AU) *per* biomass unit. The data were processed using the Origin 81 software (OriginLab Corporation, Northampton, MA, USA). The fluorescence of the soluble and insoluble fractions of the mScarlet protein was measured in the same conditions described previously.

### 2.6 SDS-PAGE and western blot analysis

To analyze the protein profile by SDS-PAGE, about 0,75 mg cell pellets recovered at the end of the growths in planktonic or static conditions by centrifugation (13′000 rpm for 20 min at 4 °C) were solubilized in 60 μL of Laemmli buffer. Then, the total cellular extracts were boiled at 95 °C for 20 min, quickly cooled on ice for 5 min, and centrifuged at 13′000 rpm for 5 min at room temperature (RT). 1 μL of samples were loaded on SDS-PAGE gel and analyzed by anti-His Western blot.

For the solubility analysis of the mScarlet protein, 45 μL of soluble or insoluble fractions were recovered and diluted in 15 μL Laemmli buffer. Then, fractions were boiled at 95 °C for different times (5 min for the soluble sample and 10 min for the insoluble fraction), quickly cooled on ice for 5 min, and finally centrifuged at 13′000 rpm for 5 min at RT. 10 μL of samples were analyzed by SDS-PAGE and anti-His Western blot. For electroblotting, the Trans-Blot Turbo Transfer System (Bio-Rad, Hercules, CA, USA) with nitrocellulose membranes was used employing the mixed molecular weight setting. After the transfer, the membrane was blocked with PBS and 5 % (w/v) milk for 1 h. Then, Monoclonal Anti-polyHistidine-Peroxidase clone HIS-1 antibody (A7058, Merck, Darmstadt, Germany) was diluted 1:2000 in PBS, 0.05 % (v/v) Tween 20, and 5 % (w/v) milk. After 1 h of incubation at RT with the antibody, the membrane was washed with PBS, 0.05 % Tween 20 three times (5 min each) and the antibody was detected with the ECL method (Cyanagen, Bologna, Italy).

### 2.7 Biofilm formation assay

The wells of a sterile 24-well flat-bottomed polystyrene plate were filled with 1 mL of the medium with a suitable dilution of the bacterial culture in the exponential growth phase (0.2 OD_600nm_) and incubated at 15 °C. After rinsing with PBS, the adherent cells were stained with 0.1 % (w/v) crystal violet, rinsed twice with double-distilled water, and thoroughly dried. Subsequently, the dye bound to the adherent cells was solubilized with 20 % (v/v) glacial acetic acid and 80 % (v/v) ethanol. After 10 min of incubation at RT, the total biofilm biomass in each well was spectrophotometrically quantified at 590 nm. Each data point was composed of three independent samples.

### 2.8 Confocal laser scanning microscopy

For the confocal microscopy analysis, the evaluation of fluorescent protein production in biofilm was performed on Nunc™ Lab-Tek® 8-well Chamber Slides (n° 177,445; Thermo Scientific, Ottawa, ON, Canada) used to grow the recombinant strains. All the microscopic observations and image acquisitions were performed with a confocal laser scanning microscope (CLSM) (LSM700-Zeiss, Germany) equipped with an Ar laser (488 nm), and a He–Ne laser (555 nm). The Z-stacks (XYZ isosurface) were obtained by driving the microscope to a point just out of focus on both the top and bottom of the biofilms with a step size of 1 μm. The images and z-stack were obtained using a 20X NA 0.8 objective. All images were analyzed with ZEN Black Imaging Software 3.0 (ZEISS, Jena, Germany) and recorded as a series of tif files with a file depth of 16 bits. The COMSTAT software package [52] was used to determine the biovolume (μm^3^/μm^2^). Biovolume provides an estimate of the biomass producing the fluorescent proteins for each condition, and two independent biofilm samples were used.

For the investigation of the effect of the induction time on GFP production, 300 μL of a suitable dilution of KrPL *LacY* + pAT-*gfp* (0.2 OD_600nm_) in the GG medium supplemented with chloramphenicol (25 μg/mL) was added to each well of a sterile Chamber Slide. IPTG (5 mM) was added at three different induction times: at the beginning (0 h), after 24 h, and after 48 h from the incubation. Uninduced cultures were used as a negative control. The plates were incubated for 96 h at 15 °C in static condition. Then, the plates were rinsed with filter-sterilized PBS and microscopic observations and image acquisitions were performed using an excitation/emission wavelength of 480/500 nm.

For the analyses of mScarlet production in biofilm under optimized conditions, 300 μL KrPL *LacY* + pAT-*mScarlet* (0.1 OD_600nm_) in the 5/5 GG medium without antibiotics and supplemented with IPTG (5 mM) were added to each well of a sterile Chamber Slide. Uninduced cultures were used as a negative control. The plates were incubated for 96 h at 15 °C in static conditions. After the incubation, the plates were rinsed with filter-sterilized PBS (as previously described) and all microscopic observations and image acquisitions were performed. The excitation/emission maxima for mScarlet are approximately 450/610 nm. The same protocol was carried out to evaluate the mScarlet production in biofilm using the recombinant strains KrPL pAT_2620-*mScarlet*, KrPL pAT_2621-*mScarlet*, and KrPL pAT_2690-*mScarlet*.

The relative percentage of fluorescent cells was evaluated with Calcofluor white stain (CFW), this dye is commonly used to reveal chitin and cellulose. The bacterial culture was prepared as described above and incubated for 96 h at 15 °C. After rinsing with filter-sterilized PBS, the well of the chamber slide was filled with 300 μl of working solution of Calcofluor white stain (CFW) and incubated for 20–30 min at RT, protected from light. All excess stain was removed by rinsing gently with filter-sterilized PBS. All microscopic observations and Z-stack acquisitions were performed. The excitation/emission maxima for Calcofluor white is approximately 453/433 nm.

### 2.9 Statistics and reproducibility of results

Statistical analyses were performed using two-tailed Student's *t-test* or two-way ANOVA with either Tukey's *post hoc* correction for multiple comparisons. *P values* of ≤0.05 were considered significant. All assays were performed at least in triplicates, and all the results were reported as a mean ± standard deviation (SD). GraphPad Prism software (Version 8, GraphPad Prism Software Inc., La Jolla, California) was used for the analyses.

### 2.10 Bioinformatic analysis

The identification of the putative promoters upstream of the selected genes (*PSHAa2620*, PSHAa2621, and PSHAa2690) in the *Ph*TAC125 genome was performed using BPROM (SoftBerry), a bacterial promoter prediction program (http://www.molquest.com).

The collected CLSM Z-stack images (saved as OME-TIFF) were analyzed with COMSTAT software package a plugin (Comstat2) to ImageJ.

## 3 Results

### 3.1 Production of the GFP and mScarlet proteins in *Ph*TAC125 biofilm

**Fig. S2:**
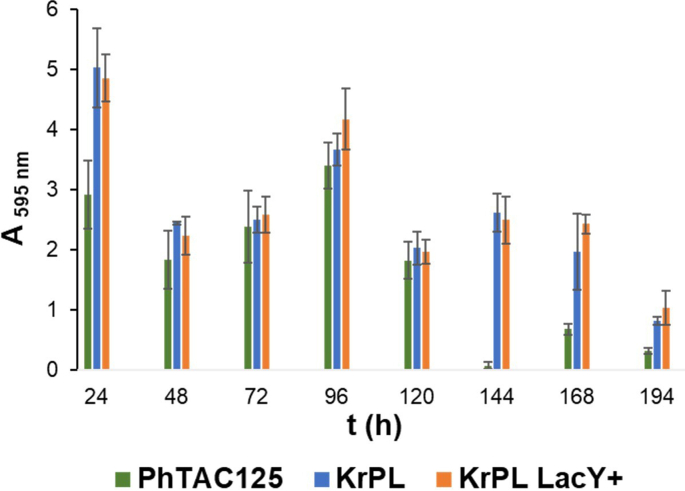
Biofilm formation kinetic of PhTAC125, KrPL, and KrPL LacY + strains.

Preliminary experiments were carried out to compare the biofilm formation ability of *Ph*TAC125 (the wild-type strain), KrPL (a *Ph*TAC125 strain devoid of the endogenous plasmid pMtBL [11]), and KrPL *LacY*^*+*^ (an engineered *Ph*TAC125 strain expressing a lactose permease to import IPTG and a truncated form of Lon protease [11]) in the GG medium at 15 °C. The tested strains showed similar biofilm formation kinetic even though a slightly higher biofilm production was observed in KrPL and KrPL *LacY*^*+*^ (Fig. S2). Due to KrPL *LacY* + well-known abilities as an unconventional host for high-quality protein production [11] it was chosen as a cell-factory to start the testing of recombinant proteins production in biofilm. The performance of this biofilm expression platform was preliminary evaluated by producing the GFP protein by the pAT vector (for details see material and methods section and Fig. S1), a derivative of the IPTG-inducible plasmid pP79 [11] consisting of a high copy replication origin, named B40-OriR [40]. This new expression plasmid conferred production yield improvements in the psychrophilic cells with respect to the pP79 vector mainly by the introduction of a high-copy number of origin replication, named B40-OriR [40].

**Fig. 1:**
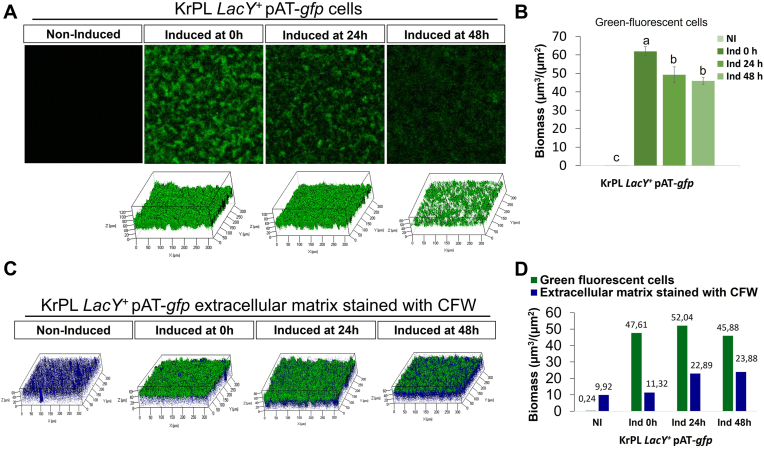
CLSM analysis of GFP protein production in biofilm at 15 °C in the GG medium for 96 h. **A** The recombinant strain, KrPL *LacY* + pAT-*gfp* was induced with 5 mM IPTG at three different times: at the beginning (Ind 0 h), after 24 h (Ind 24 h) or 48 h (Ind 48 h) of sessile growth and analyzed by CLSM after 96 h. Non-induced (NI) strains were used as a control. **B** COMSTAT quantitative analysis of biomass producing GFP in the induced cells at different times (0 h, 24 h, and 48 h from the beginning of the growth); Statistical significance was determined using two-way ANOVA followed by Tukey's *post hoc* test. Data with different letters (**a**–**c**) are significantly different (*p value* < 0.05), while those with the same letter are not significative (*p* value > 0.05) **C** Comparative analysis of EPS matrix of the biofilm (in blue) and cells producing the fluorescent protein (in green) in different conditions. Z-stack biofilm structures were obtained using the Calcofluor White Stain (CFW); **D** COMSTAT quantitative analysis of EPS matrix of the biofilm (stained with calcofluor) and amount of recombinant producing cells (green-fluorescent cells) at different induction times. (For interpretation of the references to colour in this figure legend, the reader is referred to the Web version of this article.)

**Fig. S3:**
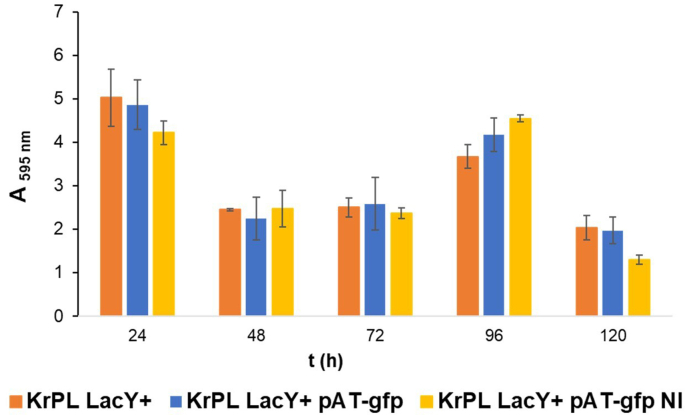
Effect of inducer and antibiotic presence on the biofilm formation.

**Fig. S4:**
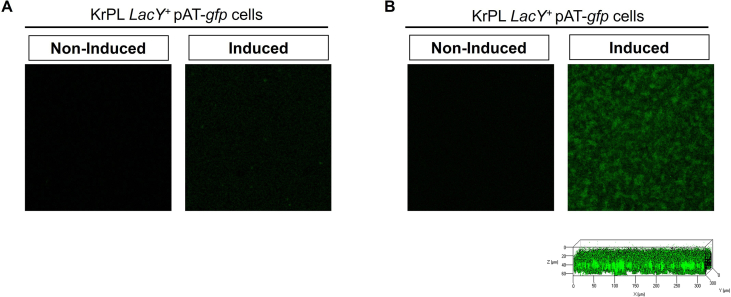
CLSM analysis of mature GFP production in biofilm at 15 °C in the GG medium.

To evaluate the influence of inducer (IPTG), and antibiotic (chloramphenicol) on the biofilm formation, the first experiments were performed growing the recombinant strain, KrPL *LacY* + pAT-*gfp*, in the GG medium supplemented with chloramphenicol (25 μg/mL) and IPTG (5 mM) in static conditions at 15 °C. As shown in Fig. S3, the presence of the inducer and the selective agent didn't influence the biofilm formation of the recombinant strain compared to that of the wild-type KrPL *LacY*^*+*^ and higher biofilm production was observed after 24 h and 96 h (Fig. S3), these two conditions were chosen to investigate the production of the green-fluorescent protein using confocal laser scanning microscopy (CLSM). The CLSM revealed a higher fluorescence recombinant production in the biofilm cells recovered after 96 h from the inoculum (Fig. S4). Then the optimal induction time was assessed by adding 5 mM IPTG at three different times of the growth (0 h, 24 h, and 48 h) and evaluating the GFP production by CLSM on the biofilm recovered after 96 h of growth. The CLSM analysis highlighted that higher GFP fluorescence was obtained by inducing the recombinant expression at the beginning of the growth (0 h) (Fig. 1A). This data was also confirmed by determining the biomass-producing mature GFP by COMSTAT [52] image analysis (Fig. 1B). Furthermore, the CLSM analysis of biofilm structure (Fig. 1C) highlighted that the GFP fluorescence is higher in the cells exposed to the air-liquid interface (in green) than the ones present in the deep layers of the biofilm matrix (in blue). This phenomenon could be related to the higher oxygen concentration at the air-liquid interface; indeed, oxygen presence is essential for GFP maturation [53]. The COMSTAT analysis confirmed that the ratio of fluorescent cells (in green) to biofilm matrix (in blue) was highest when the cells were induced at the beginning of the growth (0 h) (Fig. 1D).

**Fig. 2:**
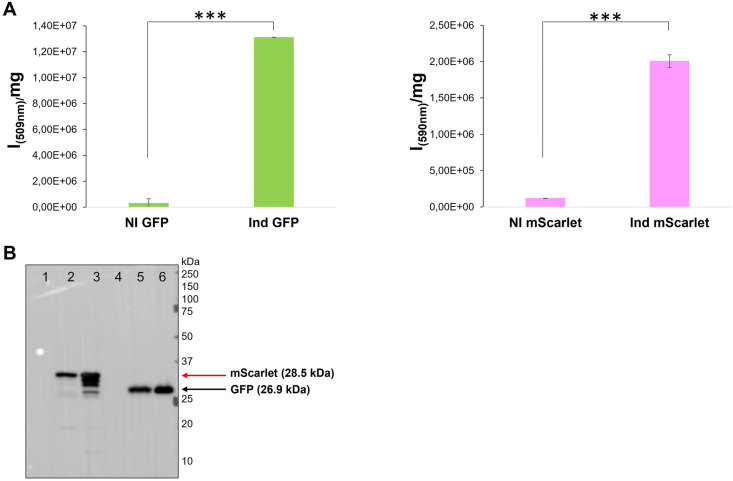
Analysis of fluorescent proteins production in biofilm. **A** Analysis of the GFP and mScarlet production by spectrofluorimetry. The fluorescence indicated on the *x*-axis was measured on non-induced and induced cells (5 mM IPTG) after 96 h from the beginning of the growth in biofilm. Fluorescence intensities are reported in arbitrary units (I) *per* biomass (mg) unit. Each data point represents the mean ± SD of three independent samples and differences were considered significant since, according to Student's *t-test*, in all conditions was *p value* < 0.05 (*p < 0.05, **p < 0.01, ***p < 0.001). **B** Analysis of GFP and mScarlet production by anti-His Western blot performed on total extracts of the KrPL *LacY* + strains grown in planktonic and sessile conditions at 15 °C. The cell extracts were examined after 24 h from the induction in planktonic condition and 96 h from the inoculum in the sessile one. Lane 1, non-induced cells in sessile condition, lane 2, induced cells producing mScarlet in sessile condition, lane 3, induced cells producing mScarlet in planktonic condition, lane 4, non-induced cells in sessile condition, lane 5, induced cells producing GFP in sessile condition, lane 6, induced cells producing GFP in planktonic condition. The same quantity of biomass was analyzed in each lane. Black and red arrows on the right of the gel represent the expected molecular weights of GFP (26.9 kDa) and mScarlet (28.5 kDa). (For interpretation of the references to colour in this figure legend, the reader is referred to the Web version of this article.)

Once induction conditions in the biofilm have been optimized, the experiments were performed also evaluating the production of another fluorescent protein, mScarlet. The pAT-*mScarlet* vector was constructed as described in material and methods section. KrPL *LacY* + pAT-*gfp* and KrPL *LacY* + pAT-*mScarlet* recombinant cells were grown by a fluidized-bed reactor using floating polystyrene supports [54]. The recombinant expression was induced with 5 mM IPTG from the beginning of the cell growth and was performed at 15 °C in static condition for 96 h. The production of the two fluorescent proteins was evaluated by spectrofluorimetric analysis (Fig. 2A). The successful production of GFP and mScarlet was further confirmed by anti-His Western blot on total cellular extracts of the recombinant KrPL *LacY* + cells (Fig. 2B). As shown in Fig. 2B the protein bands assigned to the recombinant mScarlet (28.5 kDa) and GFP (26.9 kDa) produced in the biofilm are visible in lanes 2 and 5, respectively. It's interesting to note that mScarlet produced in the planktonic condition (lane 3) was more proteolyzed than that produced in biofilm (lane 2).

### 3.2 Optimization of conditions for recombinant protein production in biofilm

**Fig. 3:**
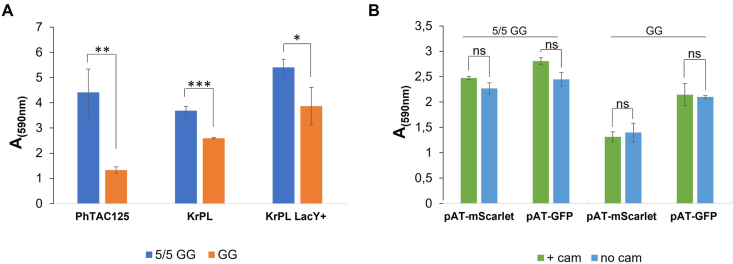
Analysis of the effect of the carbon sources concentration on biofilm formation of wild-type or antibiotics on biofilm of recombinant *Ph*TAC125 strains. **A***Ph*TAC125, KrPL, and KrPL *LacY* + biofilm obtained at 15 °C in 5/5 GG (GG with the half of carbon source) or GG. **B** KrPL *LacY* + pAT-*mScarlet* and KrPL *LacY* + pAT-*gfp* biofilm obtained at 15 °C in 5/5 GG or GG supplemented with IPTG (5 mM) in the presence (+cam) or absence (no cam) of chloramphenicol (25 μg/mL). The biofilms were analyzed after 96 h of growth with the crystal violet assay. Each data point represents the mean ± SD of three independent samples, differences were considered significant since, according to t-Student test, in all conditions was p < 0.05 (*p < 0.05, **p < 0.01, ***p < 0.001, ns: not significant). (For interpretation of the references to colour in this figure legend, the reader is referred to the Web version of this article.)

To reduce the process cost and increase biofilm production, the biofilm biomass of *Ph*TAC125 strains was evaluated in the presence of a reduced concentration of carbon sources. *Ph*TAC125, KrPL, and KrPL *LacY*^*+*^ were grown in GG with the half of carbon sources (named 5/5 GG), in static conditions for 96 h at 15 °C. The biofilm biomass was assessed by crystal violet demonstrating that the reduction of carbon sources increased the biofilm production (Fig. 3A). In addition, the influence of the antibiotic presence on biofilm biomasses produced in this new medium was explored (Fig. 3B) by growing the recombinant strains without chloramphenicol. The biofilm production resulted to be not influenced by the presence of the selective agent.

**Fig. 4:**
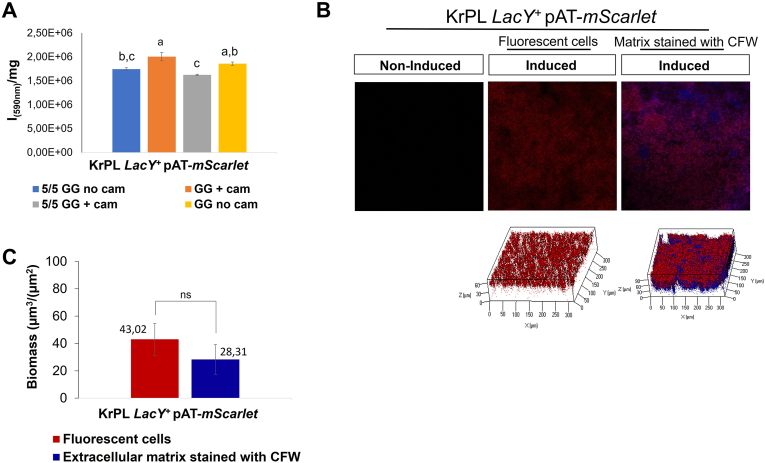
Analysis of mScarlet production in 5/5 GG under non-selective conditions. **A** Analysis of the mScarlet expression by spectrofluorimetry. The fluorescence of the protein indicated on the x-axis was monitored on induced cells (5 mM IPTG) grown in 5/5 GG without chloramphenicol, GG plus chloramphenicol (25 μg/mL), 5/5 GG plus chloramphenicol (25 μg/mL) and GG without chloramphenicol, after 96 h from the beginning of the biofilm growth. Fluorescence intensities are reported in arbitrary units (I) *per* biomass (mg) unit. Each data point represents the mean ± SD of three independent samples. Statistical significance was determined using two-way ANOVA followed by Tukey's *post hoc* test. Data with different letters (**a,b,c**) are significantly different (*p value* < 0.05), while those with the same letter are not significative (*p value >* 0.05) **B** Comparative analysis of biofilm biomass (in blue) and cells producing the fluorescent protein (in red) in 5/5 GG without chloramphenicol after 96 h from the beginning of the biofilm growth. Z-stack biofilm structures were obtained using the Calcofluor White Stain (CFW); **C** COMSTAT quantitative analysis of EPS matrix of the biofilm (stained with Calcofluor White Stain) (blue bar) and amount of recombinant florescent cells (red bar). Each data point represents the mean ± SD of three independent samples and differences were considered significant since, according to Student's *t-test*, in all conditions was *p value* < 0.05 (*p < 0.05, **p < 0.01, ***p < 0.001, ns: not significant). (For interpretation of the references to colour in this figure legend, the reader is referred to the Web version of this article.)

**Fig. S5:**
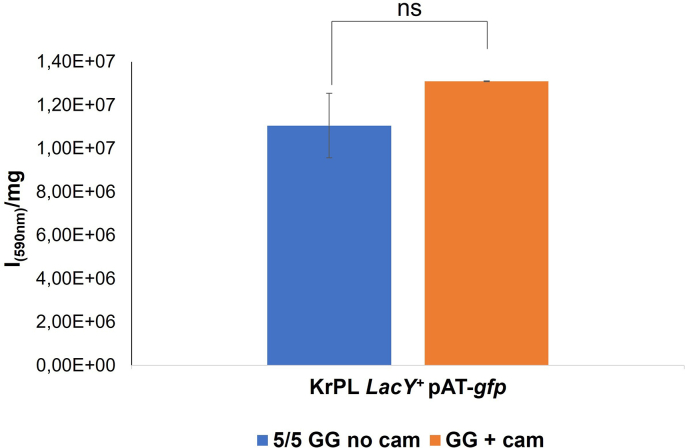
Analysis of GFP production in 5/5 GG under non-selective conditions.

To evaluate the impact of the elimination of the selective agent on the process outcomes, the recombinant protein production of mScarlet in 5/5 GG under non-selective conditions was investigated. KrPL *LacY* + pAT-*mScarlet* cells were grown in the absence of the antibiotic by a fluidized-bed reactor as previously described, and the production was compared to that obtained in GG plus chloramphenicol. As shown in Fig. 4A, the two different growth conditions resulted in almost the same fluorescence intensity *per* biomass unit, an outcome substantially identical to the GFP recombinant production (Fig. S5). The mScarlet production in 5/5 GG under a non-selective condition was also investigated using confocal laser scanning microscopy (Fig. 4B). As observed previously for GFP, mScarlet fluorescence was higher in the cells exposed to the air-liquid interface (in red) than the ones present in the deep layers of the biofilm matrix (in blue). Moreover, the COMSTAT analysis revealed that the ratio between fluorescent cells (red bar) and biofilm matrix (in blue) (Fig. 4C), indicated that most of the cells embedded in the biofilm were able to produce the mature fluorescent protein.

### 3.3 New expression systems for the recombinant protein production in biofilm

**Fig. S6:**
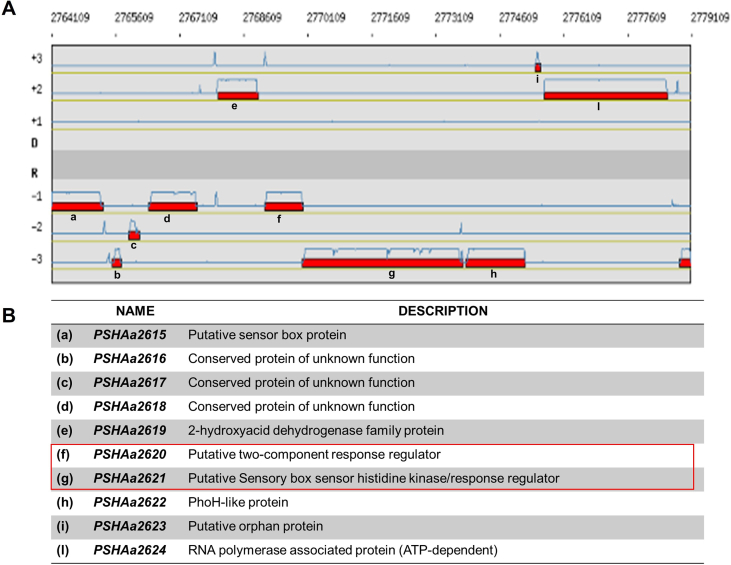
Representation of PSHAa2620 and PSHAa2621 gene locus in the *Ph*TAC125 genome.

Once the carbon source concentration has been reduced and the selective agent eliminated, a further process optimization was performed by designing heterologous gene expression systems based on biofilm-related constitutive promoters. In this way, the use of the inducer was avoided. Given the limited information on molecular mechanisms involved in *Ph*TAC125 biofilm formation, the identification of constitutive promoters was carried out by focusing on the c-di-GMP molecule [55], a second messenger that regulates the switch from planktonic to sessile growth in different bacteria [56,57]. This molecule can interact with several domains, i.e., GGDEF, EAL, or HD-GYP domains, present in many enzymes implicated in c-di-GMP synthesis or degradation [55,58]. Therefore, starting from the screening of *Ph*TAC125 genome reported by Romling and coauthors [59], a list of genes coding for putative proteins that contain such domains was drawn up (Table S2) leading us to the selection of gene PSHAa2620 and PSHAa2690. Moreover, since the PSHAa2621 gene codifies for the putative sensor histidine kinase of the *PSHAa2620-2621* two-component system (Fig. S6) it was selected too.

**Fig. 5:**
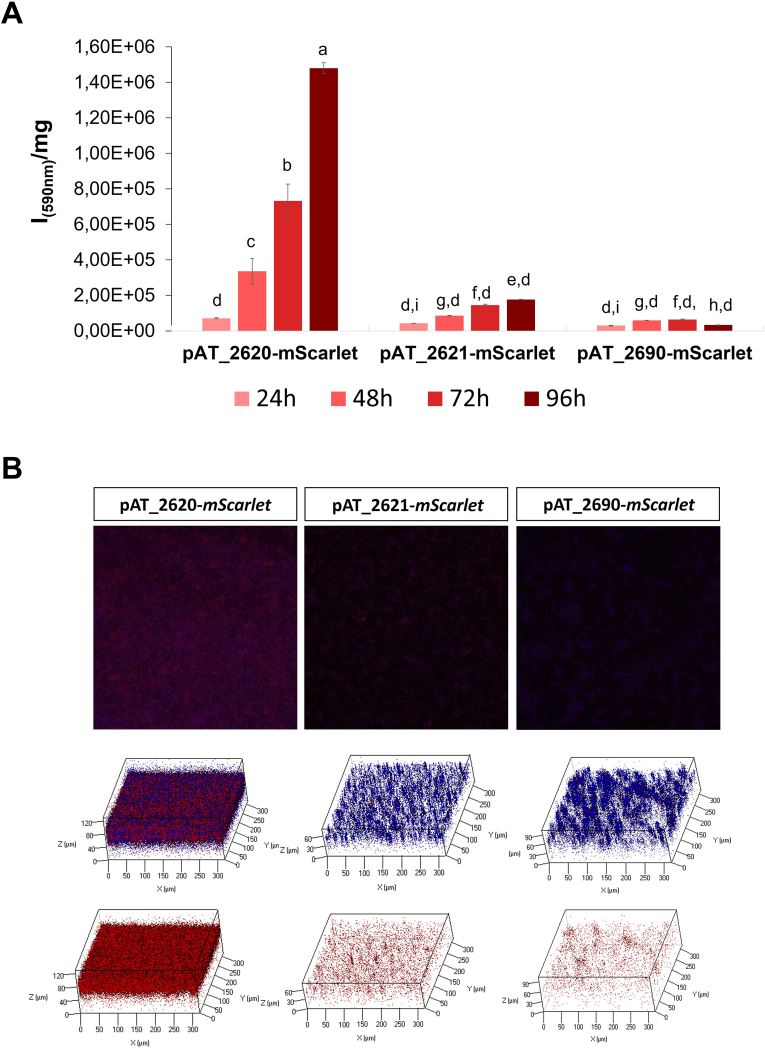
Analysis of mScarlet constitutive production in biofilm under the control of three putative biofilm-specific promoters. **A** Evaluation of the mScarlet production by spectrofluorimetry. The fluorescence of the protein indicated on the x-axis was monitored on KrPL recombinant cells after 24 h, 48 h, 72 h, and 96 h from the beginning of the growth in biofilm. Fluorescence intensities are reported in arbitrary units (I) *per* biomass (mg) unit. Each data point represents the mean ± SD of three independent samples. Statistical significance was determined using two-way ANOVA followed by Tukey's *post hoc* test. Data with different letters (**a-i**) are significantly different (*p value* < 0.05), while those with the same letter are not significative (*p* value > 0.05). **B** Comparative analysis of biofilm biomass (in blue) and cells producing the fluorescent protein (in red) in 5/5 GG after 96 h of biofilm growth. Z-stack biofilm structures were obtained using the Calcofluor White Stain (CFW) that stains the EPS matrix of the biofilm. (For interpretation of the references to colour in this figure legend, the reader is referred to the Web version of this article.)

Once selected the genes, the plasmids named pAT_2620-*mScarlet*, pAT_2621-*mScarlet*, and pAT_2690-*mScarlet* were constructed by replacing the promoter sequence of pAT-*mScarlet* with the putative promoter sequences of the PSHAa2620, PSHAa2621, and PSHAa2690 genes. The performances of the new constitutive vectors were evaluated by growing the recombinant strains, KrPL pAT_2620-*mScarlet*, KrPL pAT_2621-*mScarlet*, and KrPL pAT_2690*-mScarlet* in the 5/5 GG medium at 15 °C in non-selective and static condition for 24 h, 48 h, 72 h, and 96 h. The spectrofluorimetric analysis *per* biomass unit demonstrated the ability of all the recombinant strains to produce the reporter protein during all the stages of biofilm formation, the higher mScarlet production was obtained by using pAT_2620-*mScarlet* vector after 96 h (Fig. 5A). This result was also confirmed by CLSM image analysis (Fig. 5B).

**Fig. 6:**
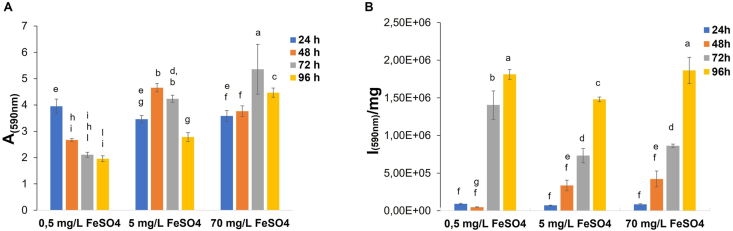
Effect of FeSO_4_ concentration on biofilm biomass and m-Scarlet production. **A** KrPL pAT_2620-*mScarlet* biofilm obtained at 15 °C in 5/5 GG supplemented with 0.5 mg/L FeSO_4_, 5 mg/L FeSO_4_ (standard concentration) or 70 mg/L FeSO_4_. The biofilms were analyzed after 24 h, 48 h, 72 h, and 96 h of growth with the crystal violet assay. Each data point represents the mean ± SD of three independent samples. Statistical significance was determined using two-way ANOVA followed by Tukey's *post hoc* test. Data with different letters (**a-l**) are significantly different (*p value* < 0.05), while those with the same letter are not significative (*p value >* 0.05). **B** Analysis of the mScarlet expression by spectrofluorimetry. The fluorescence of the protein indicated on the x-axis was monitored on KrPL recombinant cells after 24 h, 48 h, 72 h, and 96 h from the beginning of the growth in biofilm. Fluorescence intensities are reported in arbitrary units (I) *per* biomass (mg) unit. Each data point represents the mean ± SD of three independent samples. Statistical significance was determined using two-way ANOVA followed by Tukey's *post hoc* test. Data with different letters (**a-g**) are significantly different (*p value* < 0.05), while those with the same letter are not significative (*p* value > 0.05). (For interpretation of the references to colour in this figure legend, the reader is referred to the Web version of this article.)

To further improve the system, the effect of iron concentration on biofilm biomass and on the overall mScarlet production was explored. To this purpose, recombinant cells KrPL pAT_2620-*mScarlet*, were grown in 5/5 GG with 0.5 mg/L FeSO_4_, 5 mg/L FeSO_4_ or 70 mg/L FeSO_4_ in previously described conditions. The biofilm biomasses were assessed by crystal violet assay (Fig. 6A) showing that the highest concentrations of iron sulfate (70 mg/L FeSO_4_) influenced the biofilm formation of the strain increasing the biofilm production mainly after 72 h and 96 h of growth. On the other hand, it is possible to note that the mScarlet fluorescence intensity *per* biomass unit was not affected by the presence of different concentrations of iron sulfate (Fig. 6B).

**Fig. 7:**
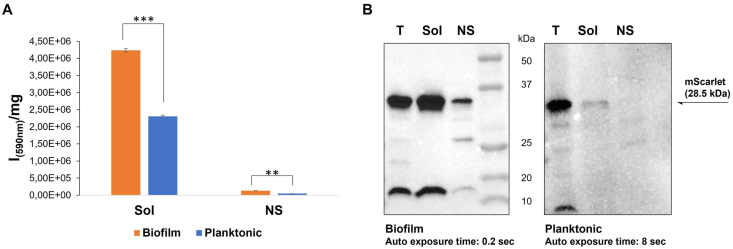
Analysis of mScarlet production on cell lysates from KrPL pAT_2620-*mScarlet* recombinant cells in biofilm or planktonic growth conditions. **A** Analysis of the mScarlet expression by spectrofluorimetry. The fluorescence of the protein indicated on the *x*-axis was monitored on soluble and insoluble fractions obtained from KrPL pAT_2620-*mScarlet* cells grown in biofilm (orange bar) and planktonic (blue bar) conditions. The fluorescence intensities are reported in arbitrary units (I) *per* total proteins concentration. **B** Comparison of the soluble and insoluble fractions after the lysis of the KrPL cells producing mScarlet in planktonic and sessile conditions. The target protein in total cellular extract (T), and soluble (Sol) and insoluble fractions (NS), recovered after lysis of biofilm cells, was detected via anti-His Western blot analysis (0.2 s auto exposure time), as shown in the left panel. The right panel represents the detection of mScarlet by anti-His Western blot analysis (8 s auto exposure time) in total cellular extract (T), and soluble (Sol) and insoluble fractions (NS) obtained after lysis of planktonic cells. For Sol in biofilm and planktonic conditions, the same quantity of the protein was analyzed. The black arrow indicates the expected molecular weights of mScarlet (28.5 kDa). (For interpretation of the references to colour in this figure legend, the reader is referred to the Web version of this article.)

KrPL pAT_2620-*mScarlet* cells were grown in planktonic and optimized biofilm conditions, then recovered cells were lysed and the production of mScarlet was evaluated by spectrofluorimetric analyses (Fig. 7A). The fluorometric analysis showed that the fluorescence intensity of the soluble fraction obtained from cells grown in biofilm was higher than that gained in planktonic cells (Fig. 7A). This result was confirmed by anti-His Western blot showing that the protein band related to mScarlet in the soluble fraction of biofilm cells is more intense than one in planktonic condition (Fig. 7B). Indeed, the signal corresponding to mScarlet produced in biofilm was detected in 0.2 s, while 8 s were necessary to observe a very weak signal in case of mScarlet produced in planktonic conditions. It's interesting to note that the low quantity of recombinant protein present in the soluble fraction obtained in planktonic condition could be also due to proteolytic events. These results suggested that the specific mScarlet production was higher in biofilms than in planktonic cells. These data were confirmed by a more accurate quantitative comparison between the mScarlet production in biofilm with the production in planktonic conditions reported in supplementary section (supplementary file 1). In the supplementary file, we conducted two comparisons. The first one involved assessing mScarlet production mediated by KrPL pAT_2620-mScarlet in cells grown in biofilm or planktonic conditions and, in this case, the production obtained in biofilm is higher. In the second comparison, we evaluated biofilm production with KrPL pAT_2620-mScarlet in biofilm against planktonic production obtained by KrPL LacY + pAT-mScarlet in conditions optimized for protein production in planktonic cultures. This second assessment was aimed to compare the biofilm production against conventional planktonic production to explore the system's potential respect to established conditions for recombinant protein production, in this case, the production resulted to be equivalent in biofilm and planktonic cells (supplementary file 1).

## 4 Discussion

To further expand the molecular tools for recombinant protein production is helpful to explore the use of unconventional approaches. These kinds of studies, although far from a coming application, are necessary to open new perspectives and find new solutions. In this paper, we explored the possibility to produce recombinant proteins in *Pseudoalteromonas haloplanktis* TAC125 biofilm. The reported skills of *Ph*TAC125 as a cell factory and as a biofilm producer inspired the idea to design an *ad hoc* expression system. The previously reported analyses on *Ph*TAC125 biofilm [44] were performed on the wild-type strain, therefore it was necessary to evaluate the ability of the two *Ph*TAC125 strains applied for the recombinant protein production to grow in biofilm. This analysis demonstrated that the capability of neither KrPL nor KrPL *LacY* + to grow in biofilm was affected by the antibiotic or IPTG. To evaluate the recombinant protein production in biofilm, two fluorescent proteins were chosen, GFP and mScarlet. We are aware that both the reporters require oxygen to maturate [51,53] and that they might not be the best choice, as biofilm is a matrix where the presence of an oxygen gradient is well-documented [25]. But, on the other hand, fluorescent proteins can be easily visualized by confocal laser scanning microscopy which is one of the best methodologies to investigate biofilm structure [60]. Indeed, by CLSM the best induction strategy was evaluated assessing that the addition of IPTG at time zero resulted to be the condition to obtain the best ratio between the fluorescent cells and the matrix biomass. On the contrary, the use of IPTG when the biofilm was already structured resulted in a not effective production (Fig. 1), likely this result is due to the well-known limitation in molecules diffusions within the biofilm [61] and/or a consequence of lack of oxygen required for maturation. Similar results were obtained by Gomes [62] and coworkers [63] that demonstrated that *E. coli* eGFP-producing biofilms were highly heterogeneous, with the cells actively producing the mature recombinant protein restricted to the top layer of the biofilm.

The production of the two fluorescent proteins in KrPL *LacY* + recombinant cells was investigated also by Western blot analysis (Fig. 2B). This experiment not only confirmed the production in biofilm conditions but also allowed an evaluation of recombinant protein quality. Indeed, mScarlet produced in biofilm condition resulted to be less affected by intracellular protease activity compared to the protein produced in planktonic conditions (Fig. 2B). A lot of papers report [25,64–66] the deep difference between planktonic and sessile cell physiology, this difference is demonstrated by several transcriptomic [67,68], proteomic [69], and metabolomic analysis. This deep difference is widely reported also in terms of extracellular proteases production [70–72], on the contrary, little information is available on the difference between intracellular protease in planktonic or sessile condition [73]. Further studies will be helpful to explore intracellular protease production in *Ph*TAC125 biofilm.

After the demonstration of the feasibility of the proposed approach, the well-known features of biofilm were exploited to reduce the process cost. The use of antibiotics is necessary for the retention of plasmid-bearing cells in planktonic [33], but the cells in biofilm tend to grow more slowly than their planktonic counterparts [74] and this behaviour justifies the increased stability of the plasmids [33]. Furthermore, a recent study [30] demonstrated that biofilm can act reserve for plasmids, allowing them to persist even under non-selective conditions. Therefore, as expected, the abolishment of the antibiotic, in our experimental conditions, didn't affect protein production (Fig. 4A). A very similar results were obtained using other hosts for the recombinant protein production in biofilm conditions [32]. It's noteworthy to underline that the resistance agent could represent up to 20 % of total proteins present in a cell [75] and its synthesis subtracts energy and precursors to the production of recombinant proteins thus the elimination of the antibiotic could be very helpful in a production process.

**Fig. S7:**
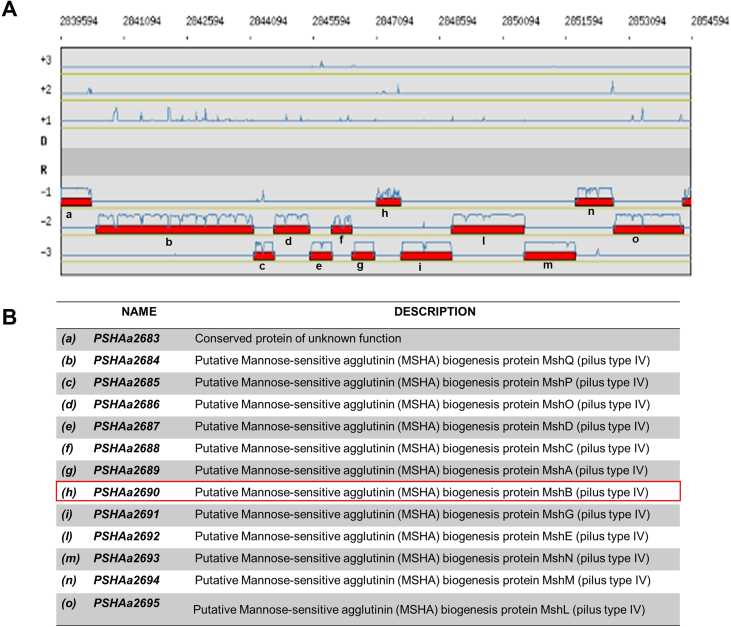
Representation of the MSHA gene cluster gene in the *Ph*TAC125 genome.

Several studies evaluated the effect of nutrient levels on biofilm formation [25, 44, 63] and some of those suggested that a high carbon source concentration inhibits biofilm formation [76,77]. Therefore, the effect of the reduction of the carbon sources was explored. The halving of the carbon sources increased the biofilm formation without affecting the fluorescent protein production. To further reduce the cost of the process it was beneficial to abolish the use of the inducer, to attain this aim it was necessary to identify a constitutive promoter suitable for the recombinant protein production in biofilm. Since no information is available on gene expression in *Ph*TAC125 grown in the sessile condition, the attention was focused on a second messenger, the c-di-GMP, that in most bacteria is involved in the switch from planktonic growth to the sessile biofilm lifestyle [59, 78, 79]. Responses to the intracellular concentration of c-di-GMP have been implicated in all phases of biofilm formation and its intracellular levels are regulated by diguanylate cyclase (DGC) and phosphodiesterase (PDE) enzymes that catalyze the synthesis and breakdown of this second messenger, respectively. DGC enzymes typically contain a GGDEF domain responsible for c-di-GMP synthesis [80], and a sensory domain that activates the DGC activity in response to external stimuli, such as nutrient concentrations [81], temperature [82], or phosphorylation [83]. On the other hand, PDE enzymes typically contain EAL or HD-GYP domains that catalyze the hydrolysis of c-di-GMP [55]. GGDEF, EAL, and HD-GYP domains, in addition to being widely conserved, are present in the vast majority of enzymes present in bacteria involved in c-di-GMP synthesis or degradation [84] but these domains have evolved to carry out also new functions. One of these functions may involve binding (but not processing) of the substrate [58]. Therefore, we screened the *Ph*TAC125 genome looking for putative genes encoding for proteins that contain GGDEF, EAL, and HD-GYP domains. The literature reported that elevated concentrations of c-di-GMP enhance surface MshA pilus production [57], therefore the genes involved in the secretion and biosynthesis of MSHA in *Ph*TAC125 were also analyzed (Fig. S7), focusing on *msh*B PSHAa2690 gene. Since MSHA gene locus is often organized into two operons [57], and usually, the promoter regulating the expression of the MSHA pilus structural subunits is located upstream of the *msh*B gene [59] we selected putative promoter the DNA region upstream of the gene upstream *msh*B (PSHAa2690 gene) to construct the pAT_2690 vector. This expression vector allowed a low recombinant protein production that is constant in all steps of biofilm development (Fig. 5).

Following this approach, a second gene PSHAa2620 was selected. This gene codifies a putative element of a two-component response system [85] involved in c-di-GMP production and containing the REC-GGDEF domain. Bacterial two-component system is composed of two proteins: the sensor histidine kinase (HK) and the response regulator (RR), this system serve to connect the detection of an environmental or intracellular signal to an appropriate response [85]. In particular, the RR protein is responsible for the execution of the specific cellular output in response to the input detected by the HK. The prototypical RR contains a conserved *N*-terminal receiver (REC) domain, which is connected to a highly variable *C*-terminal effector domain. The REC domain of the RR protein catalyses the transfer of the phosphoryl group from the associated HK onto itself, resulting in self-activation in a phosphorylation-dependent manner. Nearly 70 % of all classified RR contain a DNA-binding domain and are generally assumed to function as transcriptional regulators [86]. Of the classified RR, 8 % belong to a group that combines the REC domain with various enzymatic domains involved in signal transduction. Interestingly, a common enzymatic output domain in RR is involved in second messenger homeostasis such as cyclic diguanylate monophosphate (c-di-GMP) [86]. This finding led us to suppose that the PSHAa2620 (encoding for REC-GGDEF protein) and PSHAa2621 (encoding for the putative sensor histidine kinase, HK) genes could be constitutively expressed in biofilm. The vectors pAT_2620 and pAT_2621 were constructed using the upstream region of the selected genes and employed to produce mScarlet. The obtained data demonstrated that all plasmids allowed the production of the reporter protein although with a different extent, and the highest fluorescence *per* unit biomass was recorded after 96 h of growth (Fig. 5). Indeed, the mScarlet production is recorded in all stages of the biofilm evolution but the highest accumulation is achieved during the mature biofilm phase (Fig. 5). Moreover, the results demonstrated that pAT_2620 vector is very effective in biofilm and allows a lower production in planktonic cells. This behaviour makes pAT_2620 an *ad hoc* vector for biofilm production.

Given the importance of iron availability in biofilm formation [87–90] the effect of iron concentration on KrPL pAT_2620-mScarlet biofilm was explored. How iron affects biofilm formation varies according to the species and strain, in the case of KrPL pAT_2620-mScarlet reported results demonstrated that the presence of a higher concentration lets an increase of biofilm biomass at 96 h (Fig. 6A). The studies on the regulatory role of iron in several bacterial biofilms have revealed the complexity of this process and thus further analyses will be dedicated to uncovering the molecular mechanism by which iron controls the *Ph*TAC125 biofilm development. Notably, the iron concentration didn't affect the recombinant production of mScarlet, confirming that the pAT_2620 plasmid provided higher protein production at 96 h (Fig. 6B). This biofilm stage, in the presence of a higher concentration of iron, is characterized by a high biomass accumulation therefore this condition could guarantee a good total production yield. It's interesting to note that the more accurate quantitative analysis of the mScarlet production for cell unit (Supplementary file 1) revealed that the system for the recombinant protein production in biofilm has the same potentiality as the classical planktonic approach with some additional advantages. Indeed, it needs a lower concentration of carbon sources and doesn't require antibiotic and inducer addition.

## 5 Conclusion

In conclusion, the data reported in this paper demonstrated the feasibility of recombinant protein production in *Ph*TAC125 biofilm and allowed us to establish an optimized production strategy working on media composition and the construction of an *ad hoc* expression vector. Certainly, despite the promising preliminary results, it is necessary to evaluate the performance of the new system in the production of “difficult” proteins. Moreover, further efforts will be aimed to overcome limitations mainly related to the control of *Ph*TAC125 biofilm development and to the identification of the best processing conditions for recombinant protein production in automatic biofilm reactors.

## CRediT authorship contribution statement

**Marzia Calvanese:** Methodology, Investigation, Data curation, Conceptualization. **Caterina D'Angelo:** Methodology, Investigation, Data curation, Conceptualization. **Concetta Lauro:** Writing – review & editing, Methodology, Data curation. **Maria Luisa Tutino:** Writing – review & editing, Investigation, Formal analysis. **Ermenegilda Parrilli:** Writing – review & editing, Writing – original draft, Supervision, Methodology, Investigation, Data curation, Conceptualization.

## Declaration of competing interest

The authors declare that they have no known competing financial interests or personal relationships that could have appeared to influence the work reported in this paper.

## Data Availability

No data was used for the research described in the article.
