# Supplementary material for: Recombinant protein production in Pseudoalteromonas haloplanktis TAC125 biofilm
Source: Biofilm. 2024 Jan 24;7:100179. doi: 10.1016/j.bioflm.2024.100179 (PMC10844681; doi:10.1016/j.bioflm.2024.100179)
Supplement: Multimedia component 1 [file mmc1.docx]

| **Bacterial strains** | **Description** | **References or Source** |
| --- | --- | --- |
| *E. coli* TOP10 | [supE44, ΔlacU169 (ϕ80 lacZΔM15) hsdR17, recA1, endA1, gyrA96, thi-1, relA1] | Lab stock |
| *E. coli* S17-1*(λpir)* | thi, pro, hsd(r− m+) recA::RP4-2-TCr::Mu Kmr::Tn7 Tpr Smr λpir | Tascon et al. 1993 |
| *Ph*TAC125 | Possesses two endogenous plasmids | Médigue et al. 2005 and CIP 108707 |
| KrPL | *P. haloplanktis* TAC125 cured strain devoid of the endogenous pMtBL plasmid | Lab stock (Unpublished data) |
| KrPL *LacY^+^* | *P. haloplanktis* TAC125 variant lacks endogenous pMtBL plasmid and is resistant to kanamycin. This mutant expresses the *E. coli* lactose permease and produces a truncated version of the endogenous Lon protease | Colarusso et al. 2020 |
| **Plasmid** |  |  |
| pAT-*gfp* | High-copy number expression vector with *Ph*TAE79 lacZ regulative sequences and the Shine-Dalgarno of *trpA* (*tryptophan synthase alpha chain* encoding gene) producing GFP | This work |
| pAT-*mScarlet* | High-copy number expression vector with *Ph*TAE79 lacZ regulative sequences and the Shine-Dalgarno of *trpA* (*tryptophan synthase alpha chain* encoding gene), producing mScarlet | This work |
| pAT_2620-*mScarlet* | Vector derived from pAT-*mScarlet* containing the promoter sequence of the *PSHAa2620* gene. | This work |
| pAT_2621-*mScarlet* | Vector derived from pAT-*mScarlet* containing the promoter sequence of the *PSHAa2621* gene. | This work |
| pAT_2690-*mScarlet* | Vector derived from pAT-*mScarlet* containing the promoter sequence of the *PSHAa2690* gene. | This work |
| **Oligonucleotide** | **Sequence** |  |
| *PSHAa2620_SphI* Fw | ctc**GCATGC**GTGTTAGCGATTGACG |  |
| *PSHAa2620 NcoI* Rv | gtg**CCATGG**ATTGCCCCTAAAACG |  |
| *PSHAa2621* *SphI* Fw | cgc**GCATGC**ACACCCTAGAGGT |  |
| *PSHAa2621* *NcoI* Rv | gcg**CCATGG**GTTTGCCTGTTTT |  |
| *PSHAa2690 SphI Fw* | cgc**GCATGC**CAATGTTTGGCA |  |
| *PSHAa2690* *NcoI* Rv | gcg**CCATGG**TAATTACCTTTAATCGCT |  |
