# Supplementary material for: Recombinant protein production in Pseudoalteromonas haloplanktis TAC125 biofilm
Source: Biofilm. 2024 Jan 24;7:100179. doi: 10.1016/j.bioflm.2024.100179 (PMC10844681; doi:10.1016/j.bioflm.2024.100179)
Supplement: Multimedia component 2 [file mmc2.docx]

| **WspR family (REC-GGDEF)** | | |
| --- | --- | --- |
| *PSHAa1157* | gi\|77360100\|YP_339675 | Response regulator |
| *PSHAa2620* | gi\|77361535\|YP_341110 | Two-component response regulator |
| **PleD family (REC-REC-GGDEF)** | | |
| *PSHAa2259* | gi\|77361182\|YP_340757 | Two-component response regulator |
| **REC-PAS-GGDEF-EAL** | | |
| *PSHAa0317* | gi\|77359284\|YP_338859 | Hypothetical protein |
| **PvrR family (REC-EAL)** | | |
| *PSHAa0134* | gi\|77359106\|YP_338681 | Hypothetical protein |
| **RpfG family (REC-HD-GYP)** | | |
| *PSHAa1150* | gi\|77360093\|YP_339668 | Response regulator CheB  (Receptor modification enzyme,  protein-glutamate methylesterase) |
