# Supplementary material for: Recombinant protein production in Pseudoalteromonas haloplanktis TAC125 biofilm
Source: Biofilm. 2024 Jan 24;7:100179. doi: 10.1016/j.bioflm.2024.100179 (PMC10844681; doi:10.1016/j.bioflm.2024.100179)
Supplement: Multimedia component 3 [file mmc3.docx]

**Supplementary figure**


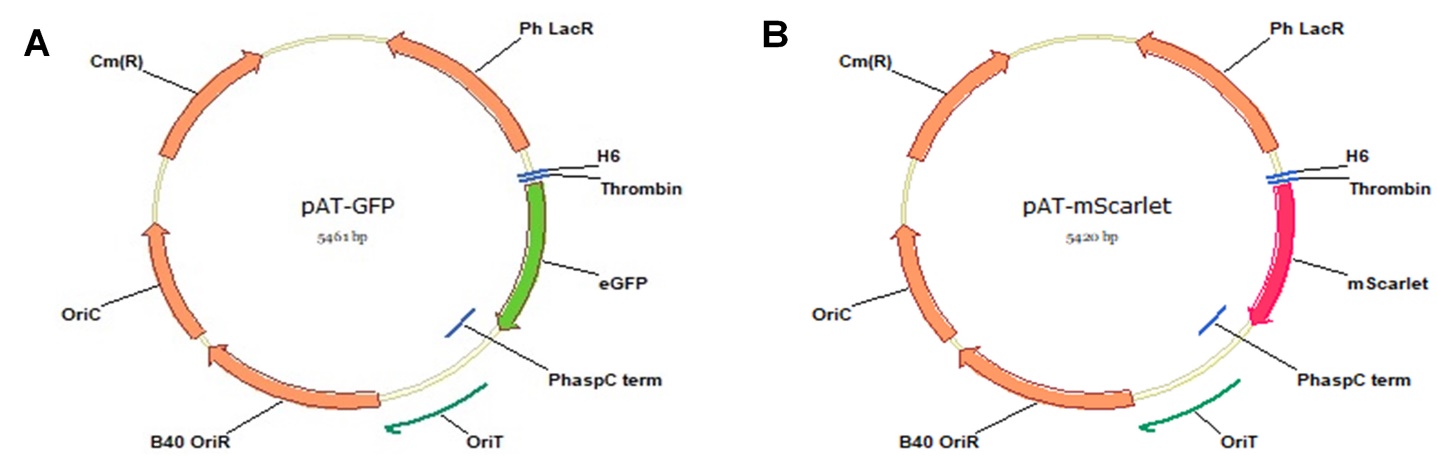


**Figure S1**: **Maps of pAT-*gfp* and pAT-*mScarlet* vectors**. In the clockwise direction, the promoter (P*lacZ*), and the regulatory sequence (*PhlacR*) of lac operon recovered from the *Ph*TAE79, 6xHis tag with Thrombin cleavage, the sequence encoding for the fluorescent protein GFP (**A**) or mScarlet (**B**),the transcriptional terminator of the *Ph*TAC125 *aspC* gene (*PhaspC* term), the conjugational DNA transfer origin (OriT), the modified the pMtBL-derived replication origin for the maintenance in *Ph*TAC125 (B40_OriR), the *E. coli* origin of replication (OriC), the coding sequences of the chloramphenicol acetyltransferase (CamR).


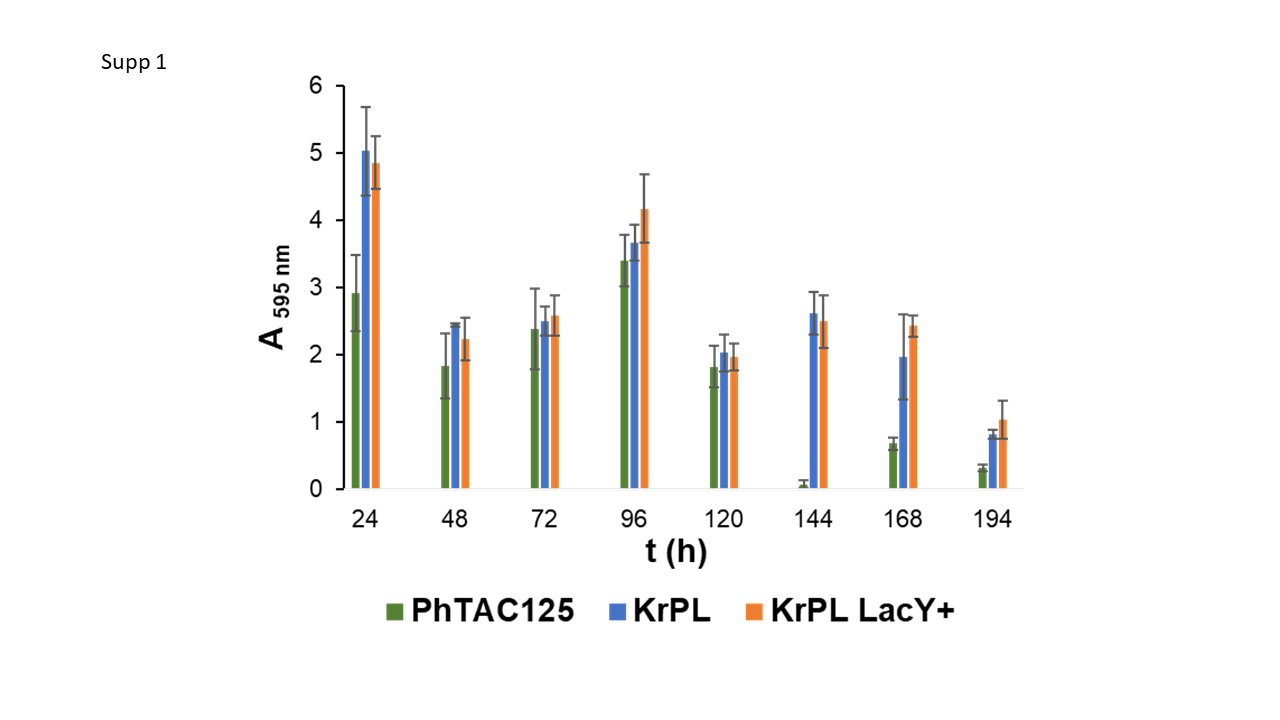


**Figure S2. Biofilm** **formation kinetic of *Ph*TAC125, KrPL, and KrPL LacY+ strains.** *Ph*TAC125, KrPL, and KrPL *LacY^+^* biofilm were obtained at 15 °C in GG medium. The biofilms were analyzed after 24 h, 48 h, 72 h, 96 h, 120 h, 144 h, 168 h and 196 h of growth with the crystal violet assay. Each data point was composed of three independent samples**.**


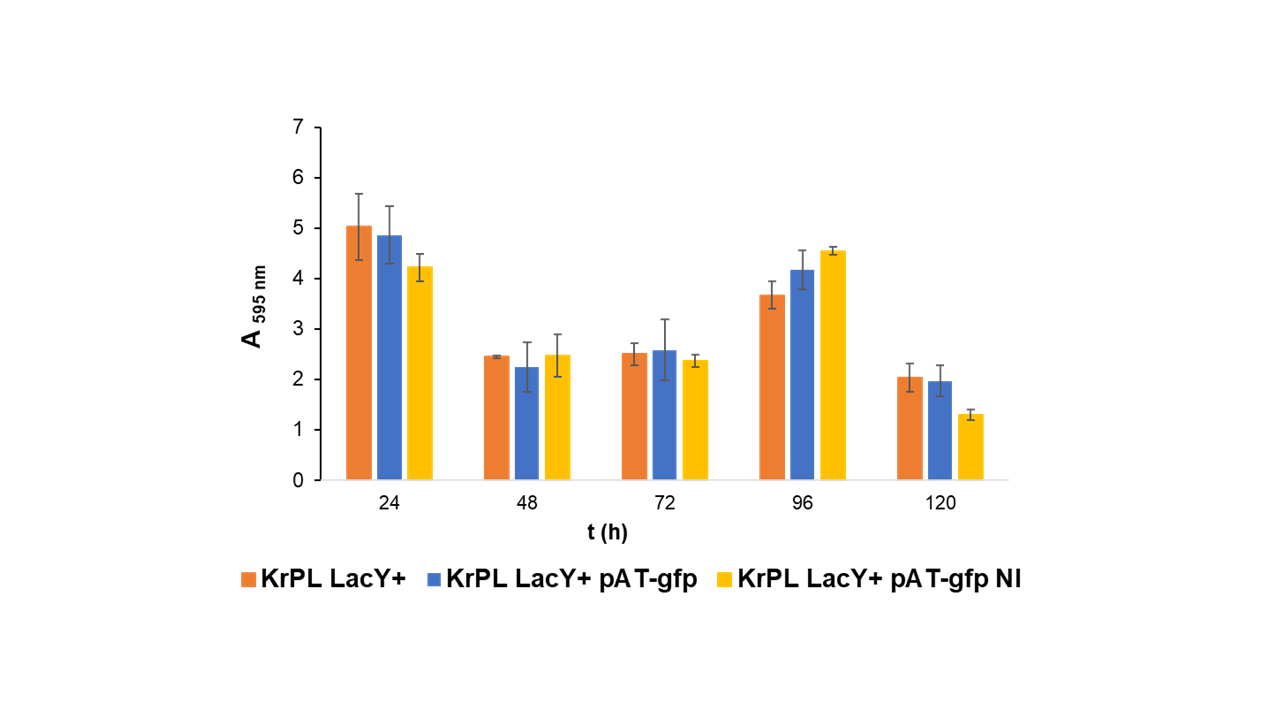


**Figure S3. Effect of inducer and antibiotic presence on the biofilm formation**. Biofilm produced in the GG medium at 15°C by wild-type KrPL *LacY^+^* (orange bar) in absence of antibiotic and IPTG compared to biofilm of recombinant strain KrPL *LacY^+^* pAT-*gfp* (blue bar) in presence of chloramphenicol (25 µg/mL) and IPTG (5mM). As a control biofilm of KrPL *LacY^+^* pAT-*gfp* non-induced (NI) strain ( yellow bar). The biofilms were analyzed after 24h, 48h, 72h, 96h, and 120h of growth with the crystal violet assay. Each data point was composed of three independent samples**.**


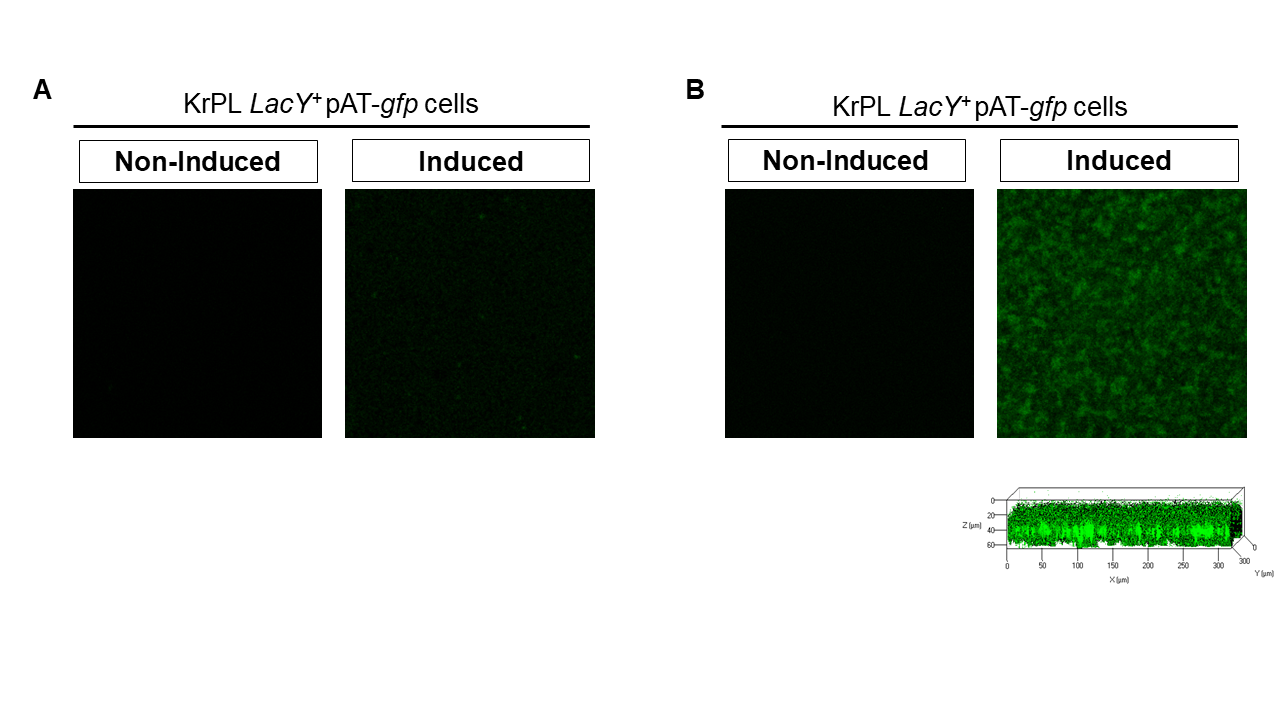


**Figure S4. CLSM analysis of GFP protein production in biofilm at 15 °C in the GG medium**. The recombinant strain, KrPL *LacY^+^* pAT-*gfp* was induced with 5 mM IPTG and the GFP production in biofilm was analyzed by CLSM after 24 h (**A**) or 96 h (**B**) of sessile growth. Non-induced strain was used as a control.


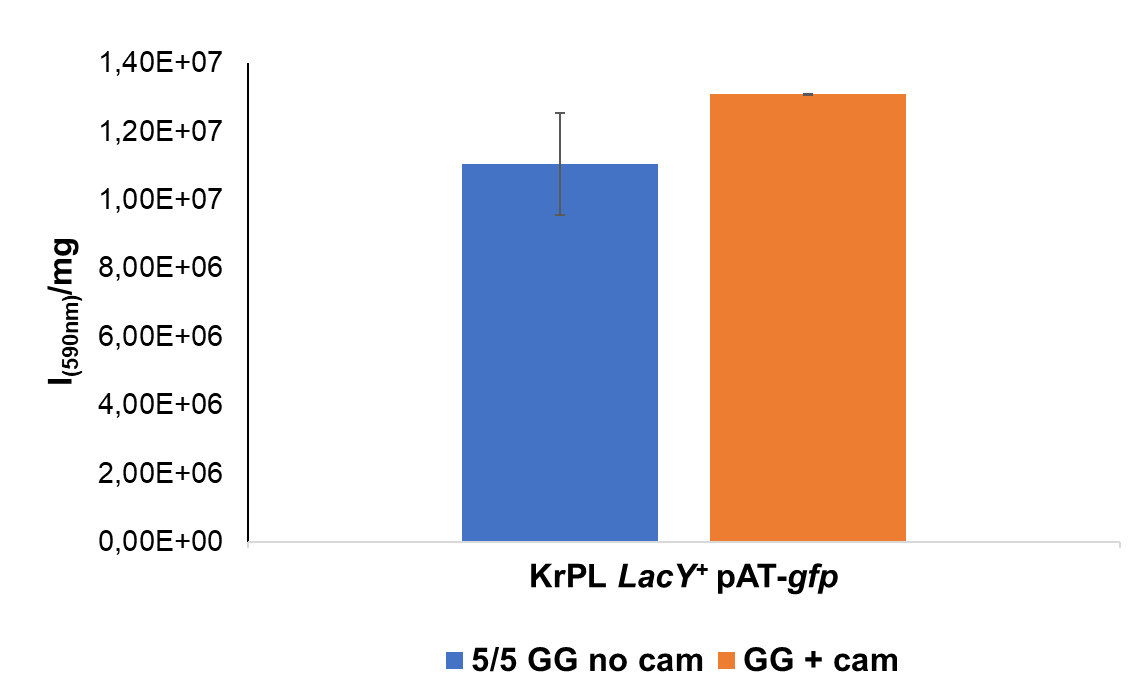


**Figure S5.** **Analysis of mature GFP production in 5/5 GG under non-selective conditions.** Analysis of the GFP expression by spectrofluorimetry. The fluorescence of the protein indicated on the x-axis was monitored on induced cells (5 mM IPTG) grown in 5/5 GG without chloramphenicol or GG plus chloramphenicol (25 µg/mL) after 96 h from the beginning of the biofilm growth. Fluorescence intensities are reported in arbitrary units (I) *per* biomass (mg) unit. Each data point represents the mean ± SD of three independent samples and differences were considered significant since, according to Student’s *t-test*, in all conditions was *p value* < 0.05 (***** *p* < 0.05, ****** *p* < 0.01, ******* *p* < 0.001).


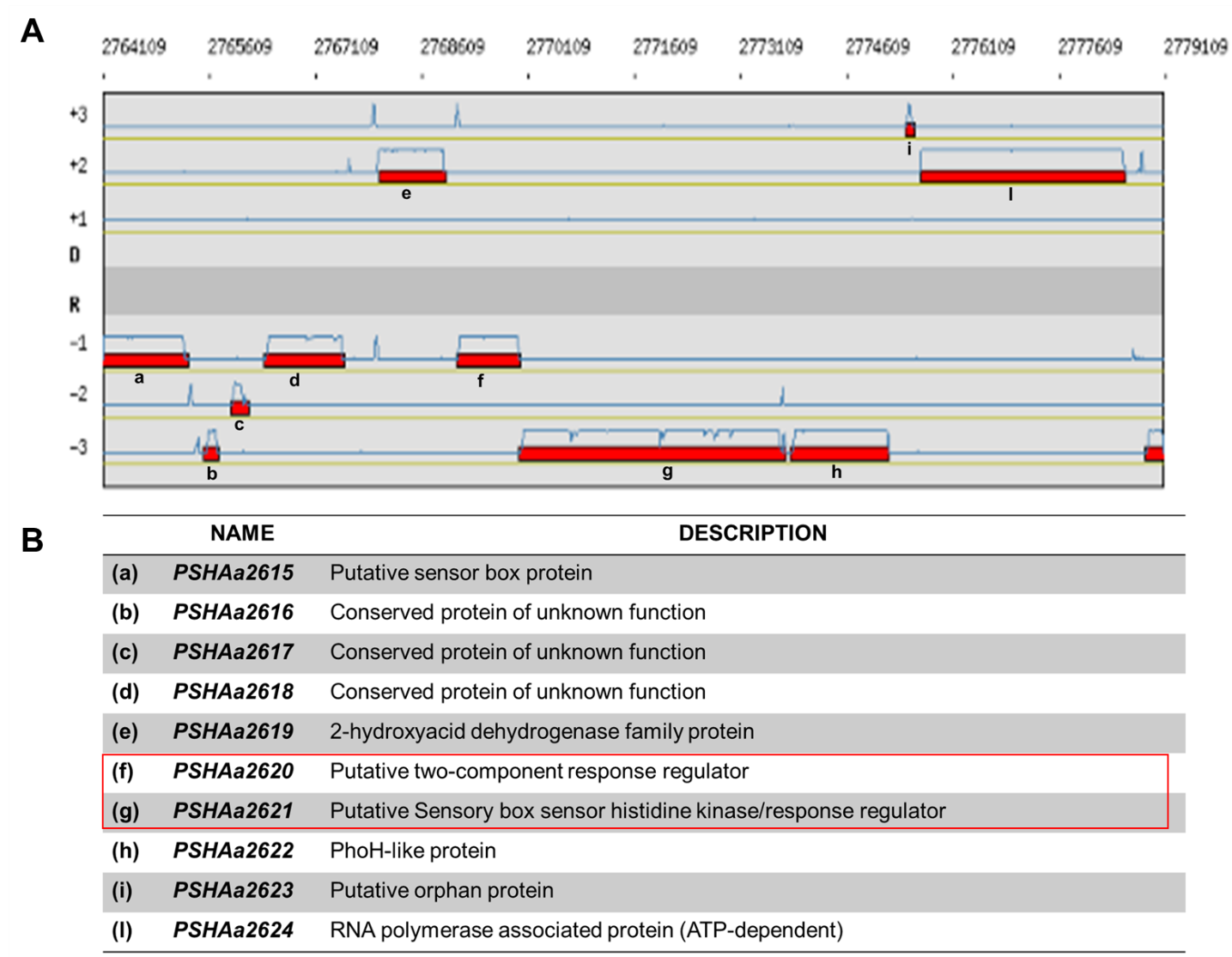


**Figure S6. Representation of PSHAa2620 and PSHAa2621 gene locus in *Ph*TAC125**.(**A**) Genomic map from MaGe and (**B**) gene description.

**
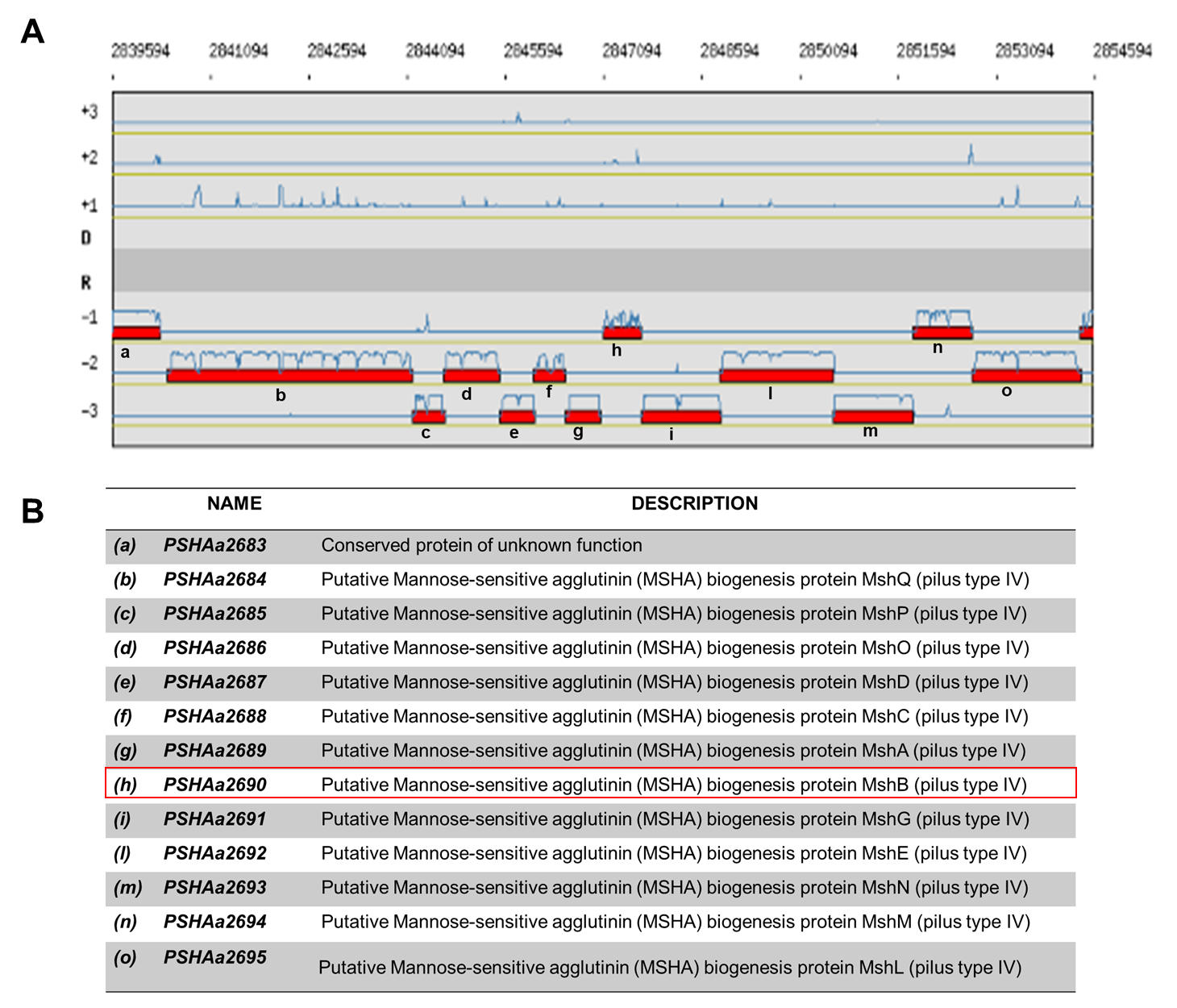
**

**Figure S7.** **Representation of the MSHA gene locus in *Ph*TAC125.** (**A**) Genomic map from MaGe and (**B**) gene description.
